# A Splendid Number

**Published:** 1872-03

**Authors:** 


### A SPLENDID NUMBER.

We think our friends will agree with us in regarding the present number of the COMPANION a good one. We give them five original articles upon subjects of interest.

We thank our contributors for their communications, and we assure them they are rendering invaluable service to the cause of science and the good of humanity. A holier, nobler work—except that of breaking the "bread of life" to perishing souls—never engaged the time and talents of human intellects.

We trust our friends will view their contributions from a higher stand-point than that of mere personal reputation, or even that of momentary advantage to science. We hope they will strive to leave a legacy of facts, from which great and enduring principles may be evolved for future generations. What the profession want are *facts*, FACTS, FACTS! Science requires these to impel it in its onward sweep in the pathway of truth.

With this view, how important to study nature as displayed in disease at the bed-side! Reports of cases wherin a strict observance of symptoms and the progressive history of the disease in its road either to health or death, with the remedies employed, and a rigid scrutiny of their therapeutical effects in efforts to arrest its destructive force, will form, in the future, the materials from which blessings are to flow to humanity.

We therefore entreat our friends everywhere to send us reports of cases. It matters not even should the style and gramatical construction of sentences in these be defective. Some of the ablest men in the profession have been held up before the gaze of the world as offenders against the rules of rhetoric and grammar, whose reputations shall endure bright and unfading, when their pugnacious critics shall lie unwept, unhonored and unsung. The only man deserving of fame and grateful remembrance is he who, even with faltering tongue and halting speech, shall leave something behind him for the good of humanity. Human suffering will not pause to inquire as to the rounded beauty of his periods, or the artistic drapery of his rhetoric; but blessings shall be heaped upon his name, and honors more enduring than those gained by the sword shall erect statues to his memory in every heart.

Therefore, we ask our friends to send in the results of their observations for the good of their brethren, and the interests of humanity.

